# Multimorbidity in patients enrolled in a community-based methadone maintenance treatment programme delivered through primary care

**DOI:** 10.15256/joc.2014.4.42

**Published:** 2014-10-06

**Authors:** Diane E. Arnold-Reed, Tom Brett, Lakkhina Troeung, Jasmine O’Neill, Rupert Backhouse, Max K. Bulsara

**Affiliations:** ^1^General Practice and Primary Health Care Research, School of Medicine, The University of Notre Dame Australia, Fremantle, WA, Australia; ^2^Fremantle Hospital, Fremantle, WA, Australia; ^3^Murray Medical Centre, Mandurah, WA, Australia; ^4^Institute for Health Research, The University of Notre Dame Australia, Fremantle, WA, Australia

**Keywords:** Multimorbidity, chronic disease, primary care, severity of illness index, methadone maintenance treatment, drug abuse, Cumulative Illness Rating Scale

## Abstract

**Background:**

Multimorbidity, the co-existence of two or more (2+) long-term conditions in an individual, is common among problem drug abusers.

**Objective:**

To delineate the patterns, multimorbidity prevalence, and disease severity in patients enrolled in a community-based primary care methadone maintenance treatment (MMT) programme.

**Design:**

This was a retrospective cohort study (*n*=274). The comparator group consisted of mainstream primary care patients. Electronic medical record assessment was performed using the Cumulative Illness Rating Scale.

**Results:**

Prevalence of multimorbidity across 2+ domains was significantly higher within the MMT sample at 88.7% (243/274) than the comparator sample at 51.8% (142/274), *p*<0.001. MMT patients were seven times more likely to have multimorbidity across 2+ domains compared with mainstream patients (OR 7.29, 95% confidence interval 4.68–11.34; *p*<0.001). Prevalence of multimorbidity was consistently high across all age groups in the MMT cohort (range 87.8–100%), while there was a positive correlation with age in the comparator cohort (*r*=0.29, *p*<0.001). Respiratory, psychiatric, and hepatic–pancreatic domains were the three most common domains with multimorbidity. Overall, MMT patients (mean±SD, 1.97±0.43) demonstrated significantly higher disease severity than mainstream patients (mean±SD, 1.18±0.78), *p<*0.001. Prevalence of moderate disease severity observed in the <45-year MMT age group was 50% higher than the ≥45-year comparator age group.

**Conclusions:**

Prevalence of multimorbidity and disease severity in MMT patients was greater than in the age- and sex-matched comparators. Patients with a history of drug abuse require co-ordinated care for treatment of their addiction, and to manage and prevent chronic illnesses. Community-based programmes delivered through primary care help fulfil this need.

## Introduction

Illicit drug use is a worldwide problem, with opioids dominating demand for the illicit drugs [1]. Problem drug abuse is associated with an increased risk of morbidity and mortality compared with the general population [2,3]. To date, most studies have focussed on the known comorbidities associated with drug abuse, such as the effects associated with non-fatal overdose [4], cognitive decline [5], hepatitis, dependence, anxiety, depression [6], and other mental health issues [7].

There is increasing evidence that multimorbidity, the co-existence of two or more long-term conditions in an individual [8], is common among problem drug abusers [9–12]. Apart from drug abuse issues, other medical complications, including mental health issues, respiratory and skin conditions, commonly found in these underserviced, marginalised populations, also need addressing [13]. Increasing multimorbidity predicts not only reduced physical functioning but also increased hospital admissions, death rates, and healthcare costs [14].

Recent research shows multimorbidity is common in substance abusers attending primary care [2], with patients more likely to accept offers of on-site integrated care for their comorbid conditions through primary care facilities, in preference to referral to specialist care [15].

Several pharmacotherapies are available for the treatment of heroin addiction [16]. Of these, methadone maintenance treatment (MMT) is one of the oldest [17], and results in better health and social outcomes for those on treatment [18]. Many current MMT clinics do not have the capacity to provide comprehensive medical care for other comorbidities present at the time of clinic visits. Community-based practice may offer an accessible alternative [15].

Our study delineates the patterns, prevalence of multimorbidity, and disease severity in a marginalised population exemplified by patients enrolled in a community-based MMT programme delivered through primary care. It is not intended to report outcomes associated with MMT.

## Materials and methods

### Setting and design

This study is a retrospective cohort study using electronic medical record review of participants attending a primary care-based MMT clinic in Western Australia. The clinic is part of a large medical centre offering comprehensive general practice care. The study population consisted of 274 patients with a history of opiate addiction attending the practice for MMT. All patients attending the clinic over a 10-year period (1 Jan 1999 to 1 Oct 2009) were included. A comparator group was selected from a cohort of age- and sex-matched patients, with no history of drug abuse, attending two mainstream general practice clinics in Perth over a 6-month period (1 Jul to 31 Dec 2008) [19].

### MMT cohort

Patients on the MMT programme were either self-referred or referred by community drug teams and other general practitioners (GPs). No information was recorded in the medical notes on the method of referral. The majority (97.1%) were polysubstance (including alcohol and smoking) abusers. Most patients (96.7%) were dependent on illicitly obtained opiates, 1.8% were dependent on prescribed opiates, and no information was available for the remaining 1.5% of patients. Once commenced on methadone by the treating GP, patients attended a designated pharmacy daily to obtain their dose of methadone. Patients were reviewed at least every 3 months once they were on a stable dose.

### Data extraction

A GP and a medically qualified researcher reviewed all patient medical records for at least 41 conditions up to the date of data extraction (Oct 2009). Each encounter was coded using the Cumulative Illness Rating Scale (CIRS) [20,21]. Unlike the published guidelines, which included “drug abuse” as part of the psychiatric domain, we nominated this as a separate category. Hence, for our purposes, scores of 0–4 were used to record the presence of disease and its severity in each of 14 domains (excluding the index condition of drug abuse). Conditions within a particular domain were noted to be present only if the information in the records suggested the condition was ongoing/chronic and then rated according to the CIRS. A total score ranging from 0 to 56 was achieved for each patient by adding maximum scores for each domain. The total score was then divided by the number of domains with morbidities to provide a severity index for each patient ranging from 0 to 4. Severity ratings were defined as 0, none/low; 1, mild; 2, moderate; and 3/4, severe [19,20].

### Definition of multimorbidity

Our definition of multimorbidity was the co-existence of two or more (2+) chronic conditions [22] in addition to drug abuse.

### Statistical analyses

Data were analysed using SPSS v22 (IBM Corporation, Armonk, NY, USA). All statistical analyses were tested against an alpha level of 0.05 (two-tailed).

Patient characteristics are expressed as means (standard deviation of the mean) for continuous variables and as frequencies for categorical variables. Patient age was calculated as age at year of data extraction (i.e. 2009). At the time of data extraction, all patients on the list were presumed to be living. The prevalence of multimorbidity was calculated as the number of patients with long-term conditions in 2+ CIRS domains as a proportion of the total sample. Severity was assessed using the CIRS Severity Index score. We also counted and compared the number of patients with at least one level 3 or 4 score across CIRS domains, as well as the number of domains with a level 3 or 4 score per patient as additional indicators of disease severity [20]. Disease patterns were examined using frequencies for the most common domain combinations across 1+, 2+, and 3+ domains. Comparisons with age- and sex-matched general practice patients were made using independent samples t-tests, chi-squared tests, and logistic regression.

Inter-rater reliability between data extractors was tested on CIRS scores and number of domains with morbidities for 40 randomly selected patients and assessed using Cronbach’s alpha.

### Ethics

Ethics approval for the study was obtained from The University of Notre Dame Australia Human Research Ethics Committee.

## Results

### Patient characteristics

Table 1 displays information on the age and sex breakdown for the MMT cohort. For the matched cohorts, 111 (40.5%) of the 274 patients were also female. The mean age of patients was 39.58±8.69 years (range 13–82 years). Female patients were generally older, with an age range of 22–82 years, compared with a range of 13–60 years for male patients, *p*=0.007. The prevalence of smoking was greater in the MMT cohort (46%, 126/274) than the comparator cohort (17.5%, 48/274), *p*<0.001.

### Inter-rater reliability

The intraclass correlation coefficient was 0.85 (95% confidence interval [CI] 0.82–0.88) for number of domains with morbidities and 0.83 (95% CI 0.79–0.86) for total CIRS scores, indicating good inter-rater reliability.

### Prevalence of multimorbidity

**Figure 1:**
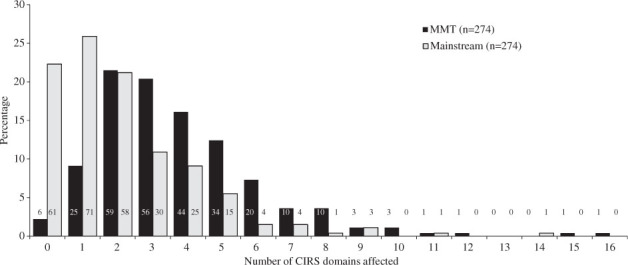
Prevalence of chronic conditions across domains in the methadone maintenance treatment (MMT) and mainstream cohorts. Numbers on the bars represent the number of patients within each group. CIRS, Cumulative Illness Rating Scale.

Figure 1 shows the prevalence of chronic conditions with reference to the number of domains. Categorised prevalence of multimorbidity across 2+ domains was significantly higher within the MMT sample at 88.7% (243/274) compared with 51.8% (142/274) in the mainstream sample (*p*<0.001). The prevalence of multimorbidity across 3+ domains was also significantly higher among MMT patients (67.2%, 184/274) than the mainstream sample (30.7%, 84/274) (*p*<0.001). In addition, 30.7% (84/274) of the MMT sample displayed multimorbidity in 5+ domains compared with 10.6% (18/274) of mainstream patients (*p*<0.001).

Logistic regression analyses showed that MMT patients were over seven times more likely to have multimorbidity across 2+ domains compared with mainstream patients (OR 7.29, 95% CI 4.68–11.34; *p*<0.001), and almost four times more likely to have multimorbidity across 3+ domains (OR 3.60, 95% CI 2.44–5.23; *p*<0.001).

**Figure 2:**
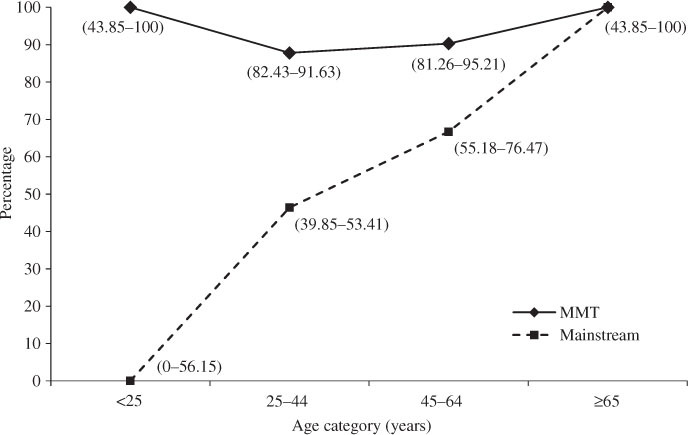
Prevalence of multimorbidity in 2+ domains according to age category in the methadone maintenance treatment (MMT) and mainstream cohorts. Symbols represent means with 95% confidence intervals in parentheses.

Figure 2 shows the prevalence of multimorbidity across 2+ domains for the MMT and comparator samples across age groups. The prevalence of multimorbidity was consistently high across all age groups in the MMT cohort ranging from 87.8% to 100%, while there was a moderate positive correlation between age and multimorbidity in the comparator sample (*r*=0.29; *p*<0.001). Moreover, the prevalence of multimorbidity was significantly higher in the MMT sample among younger patients.

### Patterns of multimorbidity

Table 2 displays the prevalence of the five most common domain combinations across 1, 2+, and 3+ domains overall and stratified by age for the MMT and comparator samples.

### Severity index

Overall, the MMT patients demonstrated significantly higher severity (mean±SD) than mainstream patients (1.97±0.43 versus 1.18±0.78; *p<*0.001). In total, 91% (249/274) of the MMT cohort were within the moderate or severe categories compared with 32.5% (89/274) of the mainstream sample (*p*<0.001).

**Figure 3:**
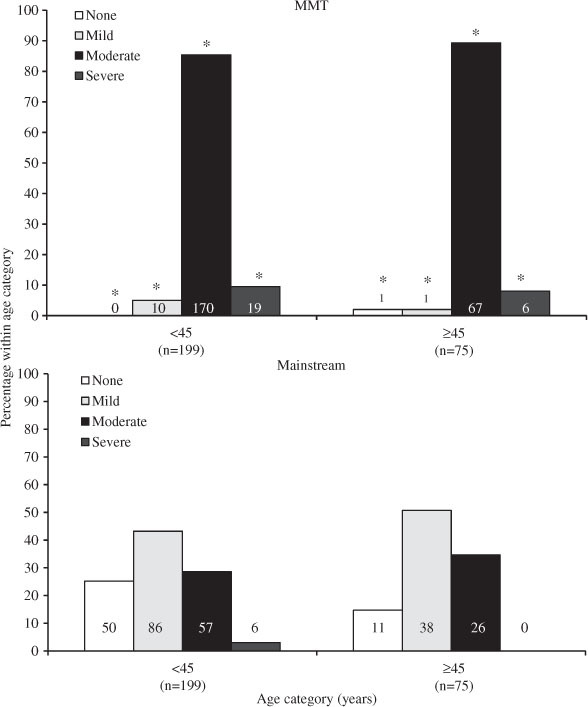
Distribution of patients according to severity index and age category in the methadone maintenance treatment (MMT) and mainstream cohorts. Numbers on the bars represent the number of patients within each group. *p<0.05 for chi-square test for MMT versus mainstream.

**Figure 4:**
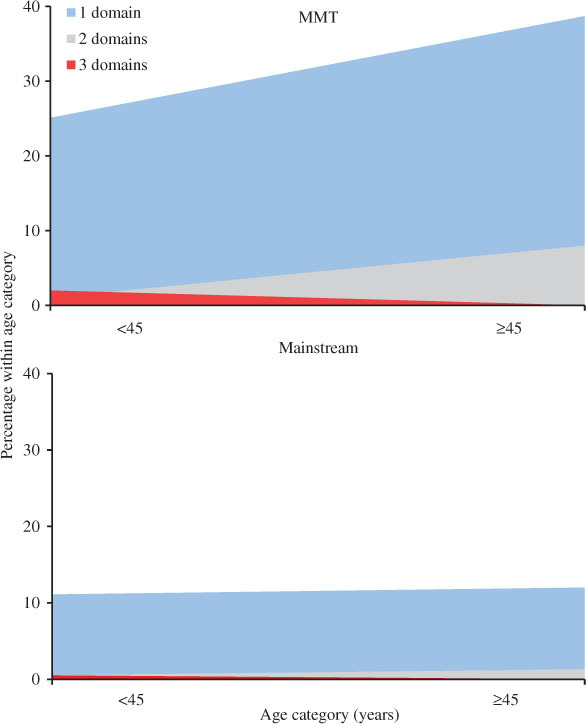
Frequency trends of number of domains with level 3 or 4 scores (moderate/severe rating) according to age category in the methadone maintenance treatment (MMT) and mainstream cohorts.

Figure 3 displays severity categories across age groups for the two samples. A greater proportion of MMT patients were represented in the moderate and severe categories than comparator patients for both the <45- and ≥45-year-old age groups. The prevalence of moderate severity observed in the <45-year-old age group of the MMT sample was 50% higher than that of the <45-year-old comparator group. In addition, 32.1% (88/274) of the MMT sample had at least one level 3 or 4 score across domains compared with 12.4% (34/274) of the mainstream cohort (*p*<0.001). Figure 4 displays the frequency trends of number of domains with level 3 or 4 scores for patients across 2+ domains for both cohorts, again revealing greater severity of multimorbidity in the MMT sample across all age groups.

## Discussion

The need for research into the multiple conditions affecting substance abusers is known [9–12]; however, focus to date has been on specific drug-related conditions [4–7], with little attention given to the cumulative and synergistic effects of these and other conditions.

This is the first study to assess 41 co-occurring conditions affecting 14 anatomical domains to estimate patterns and prevalence of multimorbidity among patients attending a designated, primary care-run, MMT service. Like our earlier mainstream practices [19] and street health study [23], we also estimated disease severity to enhance the overall picture of multimorbidity burden in this population [20].

The importance of disease burden cannot be underestimated. Government and policymakers are guided by disease distributions and population health impacts in making health service planning decisions at regional, national or international levels. Poor quality and reporting of findings means that though the global burden of disease associated with drug abuse has been identified, many studies have not been included in the comparative risk assessment, hence, the full impact is not evident [24].

Our results suggest that the prevalence of multimorbidity and the disease burden amongst the MMT group were greater than their age- and sex-matched general practice counterparts. Of the patients on MMT, 88.7% have multimorbidity and 90% have moderate to severe disease. This aligns with overseas estimates of 91% multimorbidity in a comparable cohort [2]. Results also showed that those on MMT were seven times more likely to have multimorbidity across 2+ domains and four times more likely to have multimorbidity across 3+ domains. Increased severity was reflected in the higher number and greater frequency of domains with level 3 and 4 scores.

Multimorbidity in the MMT cohort was consistently higher across age groups. This contrasts with findings from the comparator group where multimorbidity was positively correlated with age and with our [19] and other [25,26] previously published results. The S-shaped curve described by Fortin et al*.* [25] and Brett et al. [19] is replaced in the MMT cohort with a consistently high plateau distribution.

The prevalence of moderate disease severity observed in the <45-year-old MMT group was 50% higher than the ≥45-year-old comparator group and higher than that reported for a similarly aged cohort of patients attending a street health service [23]. It is inevitable that given the earlier age of onset, increased severity at an earlier age, and increased disease burden, this will result in a lifetime increase in direct cost to the health budget (specialist review, emergency department presentations, intensive care admissions) and direct and indirect costs to the economy (absenteeism, unemployment, and welfare payments).

The high prevalence of respiratory, psychiatric (not including drug abuse), and hepatic–pancreatic morbidity was not unexpected, and has been reported in other studies in drug-abusing populations [2,3,9]. Respiratory problems are known to be prevalent in mental health patients in the community [27]. Some comorbid conditions may be a direct result of drug abuse [28]. Nearly half of the MMT cohort were smokers, which may also account for the high prevalence of respiratory conditions. Similarly, increases in the respiratory, psychiatric, and musculoskeletal/skin morbidities have been reported in homeless populations [23,29]. The link between marginalisation through low socioeconomic position and multimorbidity and its early onset is also known [30–33]. The high prevalence found in the current study may thus be attributable to poor living standards or homelessness; however, socioeconomic demographics were not collected for the study.

Patients with a history of drug abuse require co-ordinated care to manage and prevent chronic illnesses. Methadone-treated patients have shown a preference to move from fragmented models of care (methadone only) to more integrated approaches where comprehensive primary medical care services are available [15,34]. Evidence shows that where drug abuse treatment and primary medical care services are more integrated, other chronic conditions are more likely to be identified [33], the adherence to treatment is higher [34], treatment benefits are better [13], and admissions to hospital and presentations to emergency departments are reduced [13,35]. Many current MMT-only clinics do not have the capacity to provide comprehensive medical care for other comorbidities present at the time of clinic visits. Primary care-based co-ordinated care may offer an accessible alternative.

Evaluation focussing on clinical outcomes of MMT services in different clinical settings highlights the importance of expanding MMT services through general practice. Not only does this mode of service delivery offer potential for maximising attendance by reducing the stigma associated with specialist treatment clinics, it also offers a more cost-effective option at a third of the cost offered through specialist psychiatric services [36].

In conclusion, our findings provide preliminary information highlighting the presence of multimorbidity in drug-affected populations presenting to a community-based primary care MMT service and which have challenging implications for the design and delivery of such healthcare services.

## Limitations

Over 47,000 Australians received a pharmacotherapy treatment in 2013, of whom 67% were treated with MMT [37]. Our study focussed on a cohort of 247 patients, which is a small representation at a national level (more especially after taking into account the systematic differences that have been reported between clinical settings [36]). Larger follow-up studies of sufficient statistical power therefore need to be undertaken at a national level to obtain the true profile of multimorbidity in this marginalised group.

With regard to age distribution, there were only three patients in each of the age categories <25 years and ≥65 years. Age stratification for the majority of analyses was undertaken on <45- and ≥45-year age groups.

Definite information on polydrug abuse or length of time on MMT was not recorded or taken into account in analyses.

Samples were drawn from different catchment areas with differences in the index of socioeconomic advantage and disadvantage. We did not directly record socioeconomic status. Estimates show that the MMT cohort had a lower ranking (7th percentile in Western Australia) compared with the comparator cohort (85–90th percentile) [38]. Whilst the MMT data were collected for patients seen over a 10-year period, the comparator mainstream practice data were collected for patients seen over 6 months.

## Figures and Tables

**Table 1:** Age and sex distribution for the methadone maintenance treatment cohort (*n*=274).

Sex	*n*	%

Female	111	40.5
Male	163	59.5

Age (years)	Mean (range)	SD

Overall	39.59 (13–82)	8.69
Male	39.50 (13–60)	8.13
Female	39.71 (22–82)	9.46

Age category (years)	*n*	%

<25	3	1.1
25–44	196	71.5
45–64	72	26.3
≥65	3	1.1

SD, standard deviation.

**Table 2:** Prevalence of the five most common domain combinations for 1, 2+ and 3+ domains overall, and stratified for age in the methadone maintenance treatment (MMT) cohort.

Number of domains	Domain	MMT % (*n*)	Mainstream practice % (*n*)	Age category (MMT only), % (*n*)			
				<25 years	25–44 years	45–64 years	≥65 years
1	Respiratory	64.6 (177)^**^	25.2 (69)	66.7 (2)	64.8 (127)	65.3 (47)	33.3 (1)
	Psychiatric (excluding drug abuse)	64.2 (176)^**^	24.1 (66)	33.3 (1)	63.8 (125)	66.7 (48)	66.7 (2)
	Hepatic–pancreatic	45.3 (124)^**^	1.5 (4)	0	39.3 (77)	65.3 (47)	0
	Musculoskeletal	39.1 (107)	31.8 (87)	33.3 (1)	34.2 (67)	50.0 (36)	66.7 (2)
	Lower gastrointestinal	25.6 (70)^**^	12.0 (33)	0	24.0 (47)	27.8 (20)	33.3 (1)
2+	Psychiatric + Respiratory	48.6 (118)^**^	9.9 (14)	33.3 (1)	42.3 (83)	45.8 (33)	33.3 (1)
	Respiratory + Hepatic–pancreatic	35.4 (86)^**^	1.4 (2)	0	28.6 (56)	41.7 (30)	0
	Psychiatric + Hepatic–pancreatic	35.4 (86)^**^	0.7 (1)	0	28.1 (55)	43.1 (31)	0
	Psychiatric + Musculoskeletal	32.5 (79)^**^	14.1 (20)	33.3 (1)	25.5 (50)	37.5 (27)	33.3 (1)
	Respiratory + Musculoskeletal	25.5 (62)	19.0 (27)	33.3 (1)	19.4 (38)	31.9 (23)	0
3+	Psychiatric + Respiratory + Hepatic–pancreatic	33.7 (62)^**^	0	0	21.4 (42)	27.8 (20)	0
	Psychiatric + Respiratory + Musculoskeletal	27.7 (51)^**^	2.4 (2)	33.3 (1)	14.8 (29)	29.2 (21)	0
	Psychiatric + Hepatic–pancreatic + Musculoskeletal	20.1 (37)^**^	1.2 (1)	0	10.7 (21)	22.2 (16)	0
	Psychiatric + Musculoskeletal + Lower gastrointestinal	16.3 (30)^*^	3.6 (3)	33.3 (1)	8.7 (17)	15.3 (11)	33.3 (1)
	Psychiatric + Respiratory + Lower gastrointestinal	15.8 (29)^*^	3.6 (3)	33.3 (1)	8.7 (17)	15.3 (11)	0

For 1 domain, denominator MMT (*n*=274), denominator mainstream (*n*=274).

For 2+ domains, denominator MMT (*n*=243), denominator mainstream (*n*=142).

For 3+ domains, denominator MMT (*n*=184), denominator mainstream (*n*=84).

^*^Chi-squared test significant at 0.05 level vs. mainstream practice.

^**^Chi-squared test significant at 0.001 level vs. mainstream practice.
